# Functional Transcomplementation between Wheat Dwarf Virus Strains in Wheat and Barley

**DOI:** 10.3390/v12010034

**Published:** 2019-12-28

**Authors:** Isabelle Abt, Marlène Souquet, Gersende Angot, Romain Mabon, Sylvie Dallot, Gaël Thébaud, Emmanuel Jacquot

**Affiliations:** 1 BGPI (Biology and Genetics of Plant-Pathogen Interactions), INRA, CIRAD, Montpellier SupAgro, University of Montpellier, Cirad TA A-54/K, Campus International de Baillarguet, 34398 Montpellier CEDEX 5, France; isaabt@yahoo.fr (I.A.); marlene.souquet@inra.fr (M.S.); gersende.angot@yahoo.fr (G.A.); romain.mabon@inra.fr (R.M.); sylvie.dallot@inra.fr (S.D.); gael.thebaud@inra.fr (G.T.); 2 Bayer CropScience, Bayer S.A.S., 16 rue Jean Marie Leclair—CS 90106, 69266 Lyon CEDEX 09, France

**Keywords:** *Mastrevirus*, host range, transcomplementation, co-inoculation

## Abstract

Wheat dwarf virus, transmitted by the leafhopper *Psammotettix alienus* in a persistent, non-propagative manner, infects numerous species from the *Poaceae* family. Data associated with wheat dwarf virus (WDV) suggest that some isolates preferentially infect wheat while other preferentially infect barley. This allowed to define the wheat strain and the barley strain. There are contradictory results in the literature regarding the ability of each of these two strains to infect its non-preferred host. To improve knowledge on the interactions between WDV strains and barley and wheat, transmission experiments were carried out using barcoded *P. alienus* and an experimental design based on single/sequential acquisitions of WDV strains and on transmissions to wheat and barley. Results showed that (I) WDV strains are transmitted with similar efficiencies by *P. alienus* males, females and larvae, (II) WDV wheat and barley strains do not infect barley and wheat plants, respectively, and (III) a functional transcomplementation between the wheat and barley strains allows a mixed infection of barley and wheat. The described ability of each WDV strain to infect a non-host plant in the presence of the other viral strain must be considered to analyze data available on WDV host range.

## 1. Introduction

Wheat dwarf disease (WDD) was described for the first time in wheat in the 1960s in the western part of the former Czechoslovak Socialist Republic (Czechoslovakia, 1918–1993) [1]. WDD is characterized by dwarfing, yellowing, mottling, streaking of leaves, suppressed heading and severe stunting of infected hosts [2], and can induce up to 90% yield losses [3,4,5]. The current distribution area of WDD includes numerous European, Middle-East, African, Western-Asian and Asian countries (Table S1). Thus, the WDD is considered as a serious problem for grain productions in most of the cereal-growing areas in the world.

Wheat dwarf virus (family *Geminiviridae*, genus *Mastrevirus*) induces the WDD on small-grain cereals. The wheat dwarf virus (WDV) genome consists of a circular, single-stranded DNA (ssDNA) of about 2.75 kb [6]. This molecule encodes four proteins: The movement protein (MP) and the coat protein (CP) which are translated from the virion-sense transcript and two replication-associated proteins (Rep and RepA), both expressed from the complementary-sense transcript.

The WDV genome also contains two non-coding sequences (the long and short intergenic regions (LIR and SIR, respectively)) which contain sequences important for viral replication and for the regulation of gene expression [7]. Phylogenetic studies carried out with complete WDV genomic sequences obtained from isolates sampled on different host species evidenced two main groups, including, respectively, the originally described wheat (noted WDV-W in this study) and barley (WDV-B in this study) strains [8,9]. The WDV genetic diversity has been further categorized into clades (A to E) based on sequence similarity and phylogenetic relationships [10]: the WDV-W group includes clades C, D and E, whereas WDV-B includes clades A (subdivided into A1 and A2 [11]) and B. WDV-B and WDV-W strains show 83–84% nucleotide identity [12], LIR and SIR being the most variable genomic regions [13]. Nucleotide identity is above 94% between WDV-B isolates and above 98% between WDV-W isolates [13,14,15,16,17].

WDV is transmitted from plant to plant in a persistent, non-propagative manner [18] by leafhoppers from the genus *Psammotettix* (Hemiptera, Cicadellidae, Deltocephalinae), a Holartic species commonly found in cereal fields and in grassland [19,20]. Morphological characteristics of these insects can be used to assign leafhoppers to the genus *Psammotettix* [21,22]. However, the accurate identification of *Psammotettix* species requires a morphological description of the male genitalia (i.e., aedeagus), which does not allow the assignment of females to the *Psammotettix* species. Due to the complex taxonomy of the *Psammotettix* spp., criteria used to identify leafhoppers species are poorly described in WDV studies. This could lead to some conflicting results, including regarding leafhopper-mediated WDV transmission experiments. Most of the published data indicate that *P. alienus* (Dahlb.) is the WDV vector [23]. However, in addition to *P. alienus*, WDV can be transmitted by *P. provincialis* (Ribaut) [24]. Molecular tools [25] or vibrational communication data [26] should be considered to accurately describe *Psammotettix* species and to improve knowledge on the role of each species in WDV epidemiology. The life cycle of *P. alienus* has been well studied [27,28]. The cycle begins with eggs laid by gravid females in the mesophyll of cereal leaves. The egg development ends with the hatching of L1 larva that evolves through five successive stages (from L1 to L5) to produce an adult (male or female). Adults and the different larvae stages were reported to be able to acquire and transmit both the wheat and barley strains of WDV [8,29,30].

WDV is able to infect a wide range of hosts belonging to the *Poaceae* family including economically important cereals, i.e., wheat, barley, oat and rye, and many wild grasses (e.g., *Apera spica-venti*, *Avena fatua*, *Lolium multiflorum* and *Poa pratensis*) [2,8,31,32]. Host ranges of WDV-W and WDV-B isolates overlap, but they seem to have wheat and barley as their preferred cereal host, respectively. Actually, the literature describing the WDV host range contains numerous contradictory reports, but suggests that both strains can infect, at least occasionally, the preferred host of the other strain. Some field surveys indicated that WDV-B isolates do not infect wheat plants [33], whereas other field surveys reported WDV-B isolates in wheat and WDV-W isolates in barley [9,12,13,16]. Leafhopper-mediated transmission experiments showed that WDV-B isolates were unable to infect wheat plants, while barley plants were infected by WDV-W isolates [8]. However, a study showed that an agro-infectious clone of WDV-B successfully infected wheat plants, in addition to barley, rye and oat [34]. Finally, WDV sequences belonging to the two strains were obtained from WDV-infected barley and wheat plants [35], highlighting that WDV-W and WDV-B can infect these two host species. Based on these different reports, it seems that the infection rate of WDV-B is low on wheat. However, when an infection of wheat by WDV-B occurs, extreme dwarfing and high mortality of plants have been reported [34,35]. Thus, several studies have already been performed on host/WDV interactions, but the ability of each WDV strain to infect wheat and barley is still unclear. To improve knowledge on WDV-B/wheat and WDV-W/barley interactions, transmission efficiencies were estimated using well-characterized *P. alienus* leafhoppers and an experimental design based on single or sequential acquisition/transmission of isolates belonging to the WDV-B and WDV-W strains. This procedure allowed us to: (I) Test the susceptibility of wheat and barley to each WDV strain, and (II) to study interactions between strains after co-inoculation of test plants. This work on the efficiency of WDV-W and WDV-B transmission to the two most economically important grain cereals is of great importance for future works on WDD epidemiology.

## 2. Materials and Methods

### 2.1. Plants, Insects and Viruses

In the whole study, the spring wheat ‘Sunstar’ [36] and the barley ‘Express’ [37] were used as host plants for both leafhoppers and viruses. Seeds were individually sown in plastic tubes containing vermiculite (for individual rearing systems and transmission experiments) or in bulks of 15–20 seeds in plastic pots (7 cm × 7 cm × 7 cm for l × d × h) containing soil (for large plastic cages rearing systems). Plants were grown at 24 °C in a temperature-controlled chamber with a light/dark period of 16/8 h and 40% RH. Seven days after sowing, i.e., at the two-leaf stage under our growth conditions, plants were used in rearing systems and in the transmission experiments.

**Figure 1:**
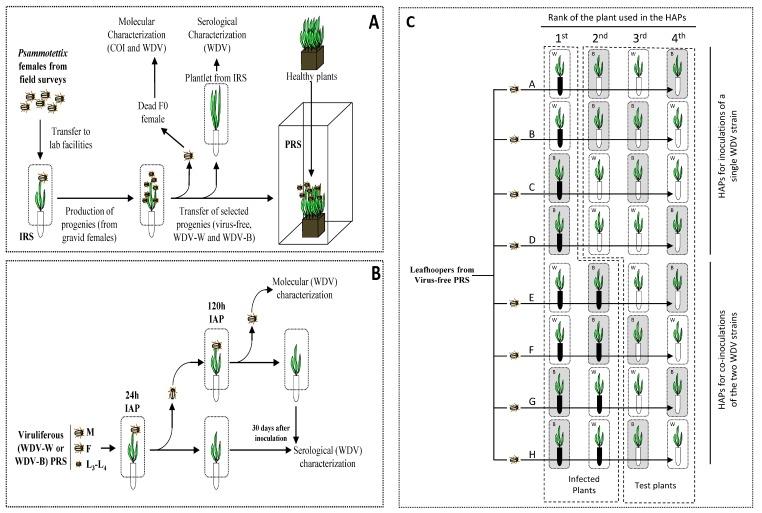
Schematic representation of the process applied to the field collected *Psammotettix* leafhoppers and of experimental procedures. (**A**) After characterization of *Psammotettix* species (COI genotyping) and viruliferous status (enzyme-linked immunosorbent assay (ELISA) and polymerase chain reaction (PCR)) of collected females, virus-free, viruliferous WDV-W and viruliferous WDV-B plastic cage rearing systems (PRS) were initiated (**panel A**). (**B**) The PRSs allow the production of viruliferous larvae (L_3_ and L_4_), males (M) and females (F) used in the characterization of transmission efficiency (**panel B**) and (**C**) virus-free males used in the study of sequential acquisition/inoculation of WDV-W and WDV-B (**panel C**). The host alternation procedures (barley plants in gray panels and wheat plants in white panels) applied to leafhoppers are presented by black arrows (HAP A to H). According to the host-alternation procedure (HAP), leafhoppers were maintained for 5 days of acquisition access period (AAP) on infected hosts (a single infected host was used for HAPs A to D and two infected hosts were used in HAPs E to H; infected hosts are represented by black tubes) and for 5 days of inoculation access period (IAP) on test plants (three test plants were used in HAPs A to D and 2 test plants were used in HAPs E to H; test plants are represented by white tubes). IRS: individual rearing system; M: leafhopper male; F: leafhopper female; L_3_–L_4_: larvae at development stage L_3_–L_4_.

Adult female leafhoppers from the genus *Psammotettix* were collected in September 2012 using a sweep net in cereal fields in the North of France (Côte-d’Or department near the city of Dijon). Each collected individual was immediately transferred on two cereal plantlets (one wheat and one barley) at the two-leaf stage, covered by a microperforated cellophane bag. These individual rearing systems (IRS) were transferred from the fields to the temperature-controlled chamber where the collected leafhoppers were maintained on their host plants (Figure 1A). Then, IRS were monitored for the presence of eggs and larvae until the female died. The sanitary status (i.e., virus-free, viruliferous for WDV-W or viruliferous for WDV-B) of field-collected (F0), female leafhoppers was ascertained using nucleic acids extraction and PCR procedures (described below). Moreover, the plantlets from IRS were tested for the presence of WDV using a serological assay (described below). A ‘healthy’ (i.e., virus-free) leafhopper *Psammotettix* population was initiated in a plastic cages rearing system (PRS) (50 cm × 50 cm × 80 cm for l × d × h) with progenies from several virus-free F0 individuals. Similarly, two strain-specific (WDV-W and WDV-B) viruliferous leafhopper *Psammotettix* populations were produced in PRS, each founded by a F1 progeny obtained from a single viruliferous gravid F0 leafhopper. To increase the number of individuals used to initiate the production of the WDV-W viruliferous leafhopper population, a few larvae were transferred from virus-free PRS to the WDV-W PRS. To maintain a large leafhopper populations in PRS, fresh plant material was added in the cages every month. The WDV-W and WDV-B viruliferous leafhopper populations were maintained on wheat and barley plants, respectively; the virus-free leafhopper *Psammotettix* population was maintained on barley. The sanitary status of the material present in the PRS was checked every other month by enzyme-linked immunosorbent assay (ELISA) and/or a polymerase chain reaction (PCR).

The isolates Enkoping1 (GenBank accession number AJ311031) and BaW1 [13] (GenBank accession number AM411651) were used as reference sequences for WDV-W and WDV-B strains, respectively.

### 2.2. DNA Extraction from Samples

Total nucleic acids were extracted from wheat and barley plantlets using the DNeasy plant Kit^®^ (Qiagen, Hilden, Germany) according to the manufacturer’s instructions. Total nucleic acids were extracted from leafhoppers using a non-destructive technique [25]. Insects were individually incubated (overnight at 55 °C) in the presence of 150 µL of TNESK buffer (Tris 50 mM pH = 7.5, NaCl 400 mM, ethylenediaminetetraacetic acid (EDTA) 20 mM, sodium dodecyl sulfate (SDS) 0.5% (*w*/*v*) and 260 µg/mL proteinase K). After a short centrifugation step (1800× *g* for 2 min at 20 °C), the supernatant was complemented with 45 µL of cold NaCl 5 M, gently homogenized and centrifuged (5000× *g* for 10 min at 4 °C). Then, 500 µL of cold absolute ethanol were added to the 195 µL fraction. The nucleic acids were pelleted by centrifugation (5000× *g* for 10 min at 4 °C), washed with 70% ethanol, dried, resuspended in 50 µL sterile water and stored at −20 °C until use.

### 2.3. Detection of WDV and Sequencing of WDV Isolates

To validate the presence of WDV in the total nucleic acid extracts, three PCR-based assays targeting WDV at the species (WDV generic detection assay) or the strain (WDV-W or WDV-B specific detection assays) levels were developed. For the WDV-generic detection assay, the genomic region corresponding to the 3′-end of the coat protein (CP) gene, the short intergenic region (SIR) and the 3′-end of the replicase gene (Rep) was amplified by PCR using 1.5 U of the GoTaq^®^ Flexi DNA Polymerase (Promega, Wisconsin, WI, USA), 0.25× Green GoTaq^®^ Flexi Buffer, 200 nM of both the forward primer WFb (5′-^809^CCACTGACATCTTTACGATGC^829^-3′, number according to GenBank accession number AJ311031) and the reverse primer WRb (5′-^1744^GGAAAGACTTCCTGGGCAAG^1725^-3′, number according to GenBank accession number AJ311031), 150 µM of dNTP, 3 mM MgCl_2_, 2 µL of total nucleic acids and adjusted with RNAse/DNAse-free water to a final volume of 50 µL. The mixture was heated 5 min at 94 °C. Then, the reactions were performed in a thermal cycler (Biometra, Goettingen, Germany) for 35 cycles of 94 °C for 30 sec, 60 °C for 1 min and 72 °C for 1 min. The run ended with an incubation step at 72 °C for 10 min. For the WDV-W strain-specific assays, the primers 1F (5′-^1398^GAGGCGAACGAGTAGTTGAT^1417^-3′, number according to GenBank accession number AJ311031) and WRb were used. Finally, the specific detection of WDV-B isolates was carried out using primers WFb and 4R (5′-^1431^AGGGTGAATCATTC^1418/1405^TTCG^1402^-3′, number according to GenBank accession number AJ311031). The WDV-B specific primer 4R overlaps the nucleotide region (nt 1406–1417) known to be deleted in WDV-B genomes. The PCR products (935 bp for the WDV species, 346 bp for the WDV-W strain and 610 bp for the WDV-B strain) were analyzed by electrophoresis in 1.5% agarose gel, stained with ethidium bromide and observed under UV illumination.

The full-length WDV genomes of WDV-w1 (accession number MN594281) and WDV-b1 (accession number MN594280) isolates maintained in PRS were amplified using primer pairs (Table S2) allowing the production of six overlapping fragments. Nucleotide sequences of PCR products were produced by Eurofins MWG Operon (Ebersberg, Germany) and analyzed using Geneious software version 10.1.2 (Biomatters Ltd., Auckland, New Zealand).

### 2.4. Characterization of Psammotettix Species Reared in PRS

To accurately characterize the *Psammotettix* species present in PRS, 27 individuals (*N* = 6, *N* = 10 and *N* = 11 for WDV-W, WDV-B and virus-free leafhopper populations, respectively) were analyzed through the cytochrome oxidase I (COI) genotyping procedure using the LCO1490 and HCO2198 primer pair [38], as previously described [25]. The COI nucleotide sequences of leafhoppers from the three PRS and 98 COI sequences from different *Psammotettix* species retrieved from GenBank (Table S3) were used to construct a phylogenetic tree using: (I) The neighbor-joining method implemented in the Geneious software with the Tamura and Nei [39] nucleotide substitution model and (II) *Macrosteles quadrilineatus* COI sequence (GenBank accession number EU981892.1) as outgroup (Figure S1).

### 2.5. Acquisition and Inoculation of WDV by P. alienus

Ten (for experiments carried out with leafhoppers viruliferous for WDV-w1 isolate) to twenty (for experiments carried out with leafhoppers viruliferous for WDV-b1 isolate) viruliferous leafhoppers were sampled from the PRS and individually transferred on 2-leaf stage plantlets for an inoculation access period (IAP) of 24 h. To prevent the escape of leafhoppers from test plants and to maintain plants under an insect-proof containment, the latter were covered by a microperforated cellophane bag during the whole experimental procedure. At the end of the IAP, the leafhoppers were individually transferred to new healthy test plants for an extra IAP of 120 h. Then, these leafhoppers were killed and test plants were maintained in the growth chamber for 4 weeks prior to the assessment of their sanitary status by serological assay (see below). This procedure was applied to WDV-w1/wheat and WDV-b1/barley combinations using larvae, male and female *P. alienus* leafhoppers as vectors.

The whole experiment is described in Figure 1B and was repeated five (for WDV-b1 transmission experiment) to 10 (for WDV-w1 transmission experiment) times (Table S4).

Male leafhoppers sampled from the virus-free PRS were used in an acquisition/inoculation procedure based on serial transfers of insects on infected/healthy hosts. In this procedure, individual leafhoppers were sequentially transferred onto four different plants. After each plant transfer, the leafhoppers were maintained on host plants for a 5-day period (i.e., 120 h). Healthy barley, healthy wheat, wheat infected by WDV-w1 and barley infected by WDV-b1, were used to design eight host-alternation procedures (HAPs) (Figure 1C, HAP A to H). At the end of the HAPs, the leafhoppers were killed and inoculated test plants were maintained in the growth chamber for 4 weeks prior to the assessment of their sanitary status using the two WDV strain-specific PCR assays. Eighty leafhoppers were used to run one replicate of the eight HAPs (i.e., 10 leafhoppers per HAP). The complete procedure of host alternations illustrated in Figure 1C was repeated six times (Table S5).

### 2.6. Serological Detection of WDV in Plants

The presence of WDV in wheat and barley plants from PRS and from transmission experiments was determined using double antibody sandwich enzyme linked immunosorbent assay (DAS-ELISA) [40]. Microtitration plates (NUNC, Maxisorp, Thermo Fisher Scientific, Massachusetts, MA, USA) were coated with 1 mg/mL of WDV antibody (DSMZ, Braunschweig, Germany) in carbonate buffer (15 mM Na_2_CO_3_, 35 mM NaHCO_3_, pH = 9.6) for 2 h at 37 °C. Between each step of the DAS-ELISA protocol, plates were washed three times with PBST buffer (PBS buffer (137 mM NaCl, 8 mM Na_2_HPO_4_, 12H_2_O, 2.7 mM KCl, 1.5 mM KH_2_PO_4_, pH = 7.4) supplemented with 0.05% (*v*/*v*) Tween 20). Plant tissue (2.5 cm of each leaf of the tested plant) was ground in a microtube containing glass balls (1 and 4 mm in diameter) in the presence of 400 µL of grinding buffer (PBST buffer supplemented with 2% (*w*/*v*) polyvinylpyrrolidone 40T). Plant sap (100 µL) was added into coated wells and left overnight at 4 °C. Alkaline phosphatase conjugated-antibody raised against WDV (DSMZ, Braunschweig, Germany), diluted according to manufacturer’s recommendation with grinding buffer supplemented with 0.2% (*w*/*v*) ovalbumin, was used as the second antibody in the DAS-ELISA procedure. After 2 h at 37 °C, washed plate wells were filled with 100 µL of p-nitrophenyl phosphate (1 mg/mL) in substrate buffer (1 N diethanolamine, pH = 9.6). After incubation at room temperature in the dark for 2 h, the absorbance at 405 nm was recorded for each well using a micro-plate reader (Multiskan FC, Thermo Fisher Scientific). A positive detection of WDV in a tested sample was considered when the OD_405_ value more than twice the OD_405_ value was obtained for healthy control samples.

### 2.7. Data Analyses

Statistical analyses were performed using R version 3.5.1 (R Core Team, 2018). The numbers of infected and healthy plants were analyzed using a generalized linear model (GLM) with binomial variance. The factors were tested by comparing nested models using a chi-square (*χ*^2^) test.

## 3. Results

### 3.1. Characterization of the Leafhoppers and WDV Isolates Used in the Experiments

The use in a phylogenetic analysis of a DNA fragment of 442 nt corresponding to nucleotides 117–558 of the *Macrosteles quadrilineatus* COI sequence allowed the assignment of 27 *Psammotettix* individuals from the different PRS (11 from the virus-free PRS, 10 from the WDV-B PRS and 6 from the WDV-W PRS) in the *Psammotettix alienus* species (Figure S1). The 10 leafhoppers sampled from the WDV-B PRS had the same COI sequence, while up to 1.6% nucleotide differences were observed among individuals from virus-free PRS. These results are in accordance with the use of a single or of several field-collected gravid females to initiate WDV-B and virus-free PRS, respectively. The different COI sequences described for individuals from the WDV-W PRS population (up to 1.6% nucleotide differences) can be explained by the transfer of insects from virus-free PRS to WDV-W PRS at the beginning of the rearing procedure.

The genome of WDV-w1 and WDV-b1 isolates were sequenced (GenBank accession MN594281 and MN594280, respectively). Phylogenetic analysis of full-length WDV sequences assigned the WDV-w1 isolate in the clade E of the wheat strain and the WDV-b1 isolate in the clade A2 of the barley strain (Figure S2). WDV-w1 and WDV-b1 genomes present 99.3% and 98.4% homologies with the reference WDV wheat strain Enk1 isolate (GenBank accession number AJ311031) and with the reference WDV barley strain BaW1 isolate (GenBank accession number AM411651), respectively (Table S6). The WDV-b1 isolate, sampled from barley in France in 2012, also presents a 23-nt insertion in the LIR region (at position 2573 of the WDV-BaW1 sequence) not reported in a previously sequenced WDV isolate. WDV-w1 and WDV-b1 share 83.6% sequence homology mostly supported by: (I) SNPs distributed along the viral genome (in coding (from 83.1% homology for CP to 87.7% homology for RepA) and non-coding (69.3% homology for LIR and 82.5% homology for SIR) sequences) and (II) the WDV-B-specific [41] 12-nt deletion (nt 1433–1444) that is also observed in the genome of WDV-b1.

Most of the SNP present in the coding sequences induce amino acid change in WDV proteins. Indeed, comparison of the WDV-w1 and WDV-b1 proteins showed 17, 32, 24 and 24 modifications of the primary amino acid sequence of MP, CP, RepA and Rep proteins, respectively.

### 3.2. Transmission of WDV by P. alienus

**Figure 2:**
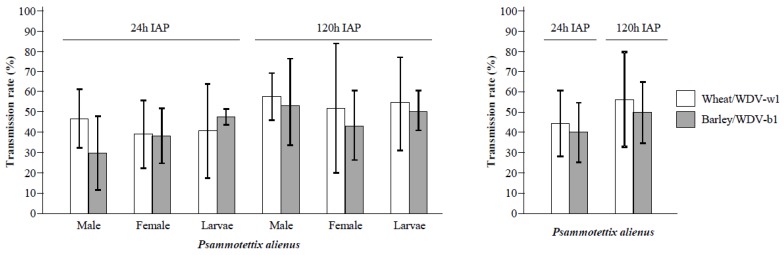
Transmission of WDV-w1 and WDV-b1 isolates by *Psammotettix alienus* males, females and larvae. Viruliferous insects (males, females and L3-L4 larvae) sampled from plastic cage rearing systems were used in transmission experiments based on 24 h and 120 h inoculation access periods (IAP). For each IAP/insect-type combination, WDV-w1 and WDV-b1 isolates were inoculated to sets of 10 wheat (WDV-w1) or 20 barley (WDV-b1) plantlets. The sanitary status of inoculated plants was determined by ELISA 4 weeks post-inoculation. Transmission rates associated with WDV-w1 and WDV-b1 are represented by white and gray bars, respectively. The whole experiment was repeated five (for WDV-b1) to ten (for WDV-w1) times. Black lines represent +/− 1 standard deviation.

Sets of 10 (for WDV-w1 experiments) to 20 (for WDV-b1 experiments) *P. alienus* males, females and larvae were sampled from the populations present in the viruliferous PRS to test their ability to transmit WDV. In this experiment, leafhoppers viruliferous for WDV-w1 and for WDV-b1 were used to inoculate wheat plants and barley plants, respectively. As populations present in the PRS were not synchronized, the age of leafhoppers used in the transmission experiments is unknown. Thus, during inoculation access periods (IAP) some adults died. To estimate virus transmission efficiencies, we considered only data associated to live insects at the end of IAPs. Moreover, experiments carried out using larvae were carefully monitored at the end of IAPs to remove from the experimental design plants with larvae that became adult during the inoculation period. This procedure removed 6% (*N* = 72) of the 1200 plants used in the experiment (Table S4). Analysis of infection rates using a GLM showed no significant differences in transmission efficiency associated (I) to *Psammotettix* males, females and larvae (*p* = 0.31) and (II) to the WDV-w1 and WDV-b1 isolates (*p* = 0.10). This indicates that the development stage, the sex of vectors and the isolates used in the experiments do not affect *P. alienus* ability to transmit WDV. However, a significant difference in virus transmission was observed between IAPs (*p* = 1.5 × 10^−4^). These transmission rates were estimated to 42.2% (95% confidence interval (CI_95%_): 38.2–49.3%) for the 24-h IAP to 53.6% (CI_95%_: 49.3–57.8%) for the 120-h IAP. This result indicates that some viruliferous leafhoppers need more than 24 h to inoculate infectious viral particle(s) to susceptible plants (Figure 2). Based on these results, the inoculation access period used in the next steps of the work was fixed to 120 h.

### 3.3. Inoculation of WDV-w1 and WDV-b1 to Barley and Wheat Plantlets

To test the ability of a WDV isolate to infect wheat and barley plants, host alternation procedures (HAPs) were designed. In these procedures, leafhopper males from the virus-free PRS were used. Males were allowed to acquire virus for a 5-day (i.e., 120 h) acquisition access period (AAP) on infected wheat and/or barley plants.

**Figure 3:**
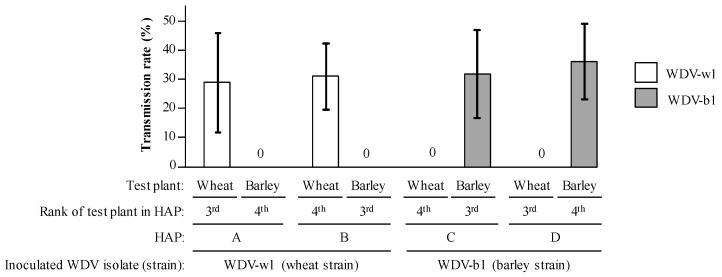
Inoculation of WDV-w1 or WDV-b1 isolates to wheat and barley plants using host alternation procedures (HAPs). Virus-free leafhoppers were allowed to acquire WDV on an infected plant for a 5-day acquisition access period (AAP). Then, according to HAP procedures A to D, leafhoppers were transferred every 5 days to a healthy wheat or barley plantlet. Ten leafhoppers were used in each HAP and the whole experiment was repeated six times. Sanitary status of third and fourth plants used in HAPs was assessed 4 weeks post-inoculation. Transmission rates associated with WDV-w1 and WDV-b1 are presented by white and gray bars, respectively. Black lines represent +/− 1 standard deviation. The HAPs are described in Figure 1C.

Then, viruliferous insects were serially transferred onto wheat and barley healthy plants according to these HAPs (Figure 1C). To improve transmission efficiencies, leafhoppers were maintained on each test plant of the HAP procedure for 120 h IAP. As previously described, only plants with live insects at the end of IAPs were considered for data analysis (Table S5). In HAPs A and B, based on the single acquisition of the WDV-w1 isolate, wheat test plants (i.e., the third (HAP A) and the fourth (HAP B) plant of the host alternation procedures, see Figure 1C) were infected at a rate of 29.0 (+/− 17.0%) and 31.1 (+/− 11.4%), respectively (Figure 3, HAPs A and B). On the contrary, none of the 95 barley plants, that correspond to the third (HAP B) and the fourth (HAP A) plants inoculated by leafhoppers viruliferous for WDV-w1, was infected at the end of the experiment. In the HAPs C and D, that corresponds to inoculation of the WDV-b1 isolate, 31.9 (+/− 15.1%) and 36.3 (+/− 13.0%) of barley test plants, which correspond to the third (HAP C) and the fourth (HAP D) plant of these host alternation procedures, were infected, respectively, while none of the 102 wheat plants inoculated by leafhoppers viruliferous for WDV-b1 in HAPs C and D were infected (Figure 1C and Figure 3, HAPs C and D). The host specificity of the WDV strains observed in this experiment suggests that WDV-w1 and WDV-b1 are not infectious on barley and wheat, respectively.

### 3.4. Transmission Efficiencies in Multiple Acquisition/Inoculation Procedures

**Figure 4:**
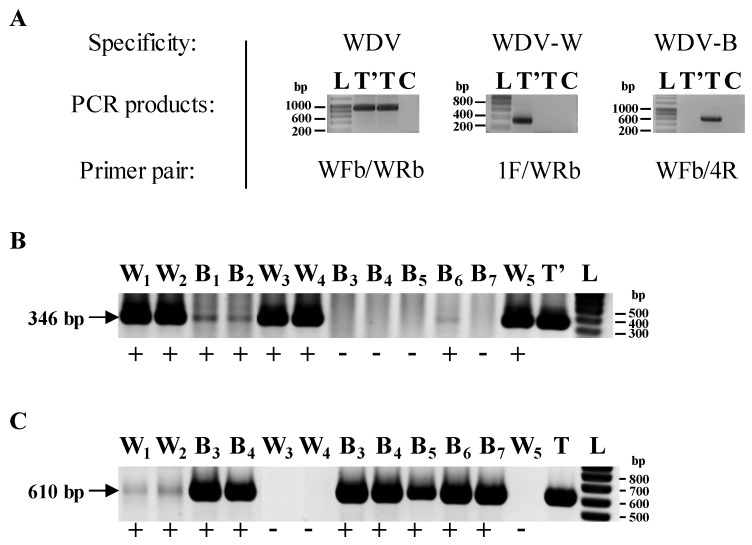
Strain-specific detection of WDV-w1 and WDV-b1 isolates. WDV species- and strain-specific PCR assays were set up using appropriate primer pairs (**A**). Controls containing WDV-W (T’), WDV-B (T) and no template control (C) were used in the three different assays. Primer pairs used in assays are listed. The presence of WDV-W and WDV-B genomes in tested samples is highlighted by the 346 bp (detection of WDV-W) and 610 bp (detection of WDV-B) fragments, respectively. Sanitary status of wheat and barley plants inoculated with leafhoppers viruliferous for both WDV-w1 and WDV-b1 was characterized using WDV-W (**B**) and WDV-B (**C**) strain-specific PCR assays. Two µL of total nucleic acids extracted from leaf fragments were used as a matrix. Arrows denote the presence of an amplified fragment. W_1_ to W_5_: five wheat test plants from HAPs; B_1_ to B_7_: seven barley test plants from HAPs; T’ and T: positive controls for WDV-W and WDV-B specific PCR assays; L: ladder.

**Figure 5:**
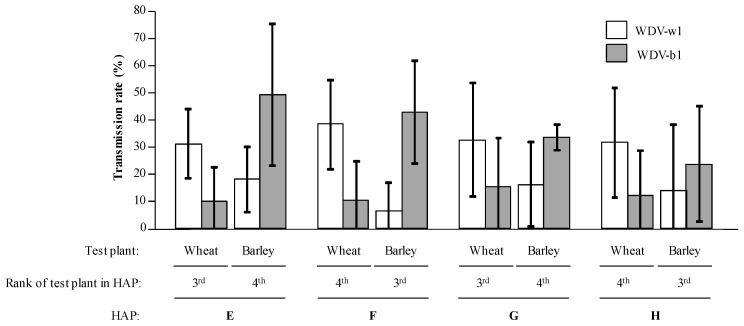
Co-inoculation of WDV-w1 and WDV-b1 isolates to wheat and barley plants using host alternation procedures (HAP). Virus-free leafhoppers were allowed to acquire both WDV-w1 and WDV-b1 isolates for a 5-day acquisition access period (AAP) on an infected wheat and for a 5-day AAP on infected barley. Then, according to HAP procedures E to H, leafhoppers were transferred every 5 days to a healthy wheat or barley plantlet. Ten leafhoppers were used in each HAP and the whole experiment was repeated six times. Sanitary status of the plants in third and fourth positions in the HAPs was determined 30 days post-inoculation using strain-specific PCR detection assays. Transmission rates associated to WDV-w1 and WDV-b1 are represented by white and gray bars, respectively. Black lines represent +/− 1 standard deviation. The HAPs are described in Figure 1C.

Host alternation procedures with sequential acquisitions of WDV-w1 and WDV-b1 were designed (Figure 1C, HAPs E to H). After two periods of 120 h spent (I) on infected wheat plants then barley plants (HAPs E and F) or (II) on barley plants then on infected wheat plants (HAPs G and H), viruliferous leafhoppers were allowed to feed on healthy barley and wheat test plants for a 120 h IAP on each host. As test plants from HAPs E to H were inoculated by leafhoppers viruliferous for the two WDV strains, we tested each inoculated plant for the presence of both WDV-w1 and WDV-b1. Thus, species- and strain-specific PCR assays were developed and used to describe the sanitary status of inoculated plants (Figure 4). WDV-w1 was detected in 31.8 (+/− 13%) to 39.1 (+/− 16.8%) in wheat plants and WDV-b1 was detected in 24.3 (+/−21.8%) to 50.4 (+/− 26.6%) in barley plants (Figure 5) which is close to results obtained from single acquisition procedures (Figure 3, HAPs A to D). The molecular detection of the barley strain WDV-b1 isolate in wheat plants (from 10.2 to 15.7%) and of the wheat strain WDV-w1 isolate in barley plants (3.5 to 18.5%) clearly indicates that these two isolates have, with HAPs E to H, the possibility to infect the host species they did not infect with HAPs A to D.

However, single infections of WDV-b1 on wheat plants and of WDV-w1 on barley plants were not detected (not shown). Indeed, the WDV-b1/wheat and WDV-w1/barley infections were all observed from WDV-b1/WDV-w1 mixed-infected plants.

## 4. Discussion

The characterization of host range, aggressiveness and transmission abilities provides important biological grounds for epidemiological studies on a pathosystem. However, the production of such data mainly depends on: (I) The sensitivity and the specificity of the tools used to detect and characterize the targeted organism (e.g., to perform field surveys) and (II) the availability of specimen(s) from each partner involved in the studied pathosystem (e.g., to preform experiments in laboratory). Indeed, for insect-borne plant viruses, well-described vector(s) and viral isolate(s) are required to set up any laboratory experiment. The first description of the wheat dwarf pathosystem dates back to 1961 [1]. Until the early 1980s, the description of this pathosystem was only based on symptom observations from naturally-infected plants (i.e., field surveys) and from leafhopper-mediated inoculations carried out in laboratories. During the past four decades, several serological and molecular tools were developed to better describe the interactions between WDV, its host plants and vector insects. However, numerous researches on WDV/host interactions did not consider viral strains, because (I) WDV strains were not yet described at the time of the work and/or (II) the sanitary status of the samples was established using tools targeting WDV at the genus level only. It is established that WDV strains have distinct but overlapping host ranges. Thus, WDV/host studies retrieved from the literature and carried out with isolates described at the species level should be considered with caution for the description of the host range of this virus.

There are important variations in vector transmission ability both between and within virus species transmitted in a persistent manner (e.g., [42,43]). Misidentification of insect specimens used in laboratories, known to be common even in museum collections [44], can strongly affect the interpretation of insect-mediated transmission experiments. Thus, leafhoppers must be accurately characterized before their use in WDV transmission studies. The leafhopper *P. alienus* species, reported in the literature to be the vector of WDV, cannot be accurately described without the use of a molecular approach (i.e., COI barcoding) [25]. Indeed, any description of species belonging to the *Psammotettix* genus using morphometric characteristics of the aedeagus is not reliable, and can lead to the assignment of leafhoppers to a wrong species. In this work, leafhoppers were characterized using a barcoding approach. According to the phylogenetic tree built from COI sequences, the *Psammotettix* individuals used in this study as WDV vectors do belong to the *P. alienus* species [25]. Characterization of viral isolates used in transmission experiments is also necessary to avoid a misinterpretation of the transmission results. The full-length genome of the WDV-w1 and WDV-b1 isolates were sequenced and compared to 27 WDV referent genomic sequences used in the most recently published phylogenetic analysis of WDV diversity [11] to accurately demonstrate that the WDV-w1 isolate belongs to the WDV-W strain and the WDV-b1 isolate belongs to the WDV-B strain. As the recent description of WDV clades has not been associated to a biological property of WDV isolates, the clade assignment of WDV-w1 (clade B) and WDV-b1 (clade A2) has not been considered in the analysis of the results. WDV-w1 and WDV-b1 isolates, maintained since their collection in PRS, accumulate in infected plants at a level above the serological diagnostic threshold within two weeks post inoculation.

Transmission experiments carried out with leafhopper males, females and larvae showed (I) no differences in the ability to transmit the two isolates and (II) contrasted results between infection rates associated to the preferred host of each isolate (mean transmission efficiency above 30%) and the non-preferred hosts (0 out of 197 inoculated plants). Thus, under our experimental conditions, the WDV-w1 and WDV-b1 isolates are not able to infect barley and wheat, respectively. However, some plants (wheat and barley) inoculated with leafhoppers viruliferous for both WDV-w1 and WDV-b1 were infected by the two viral strains. In these experiments, the two isolates were sequentially acquired by leafhoppers using source plants infected by only one of the isolates.

During the persistent transmission of the virus, the two types of particles are not supposed to exchange proteins or genetic information in the vector, as they do not replicate within the leafhopper [45]. Thus, test plants were inoculated by one type of particle, the other, or both. The absence of wheat plant infected by WDV-b1 only and of barley plant infected by WDV-w1 only is in accordance with the host specificity of WDV strains described from single acquisition/inoculation experiments (HAPs A to D). The observation of co-infected plants in HAPs E to H suggests transcomplementation of the non-infectious strain by some viral component(s) from the infectious strain (i.e., WDV-w1 in wheat and WDV-b1 in barley). This process would enable the completion at least one of the steps of the infection cycle that fails during WDV-w1/barley and WDV-b1/wheat interactions. Transcomplementation between plant viruses belonging to closely-related or phylogenetically-divergent species is a very common phenomenon. Indeed, at least 69 virus species from 35 genera can be involved in transcomplementation during these mixed infections of host plants [46]. Transcomplementation occurs when the infectious cycle of one virus is enhanced or supported by protein(s) expressed by the other virus. Thus, the helper virus can enhance or modify properties (transmission characteristics [47], cell-to-cell and long-distance movements [48,49], viral accumulation [50] and host range [51]) of the dependent virus. Nucleotide divergence (16.6%) between WDV-w1 and WDV-b1 isolates are located along the viral genome (from 11.5% nt. divergence in the Rep gene to 30.7% nt. divergence in the LIR region, Table S6). In the coding sequences of the viral genomes, 97 amino acids located in the four proteins encoded by WDV were different between the two isolates. Based on these nucleotide/protein comparisons, it is presently not possible to speculate about genomic region(s), gene(s), functional domain(s), amino acid(s) or nucleotide(s) that could be responsible for the host specificity of WDV isolates and the transcomplementation phenomenon evidenced in this work. Further work may investigate the mechanisms underlying this functional transcomplementation by the use of chimeric infectious viral clones or transgenic plants expressing parts of the helper virus genome [52].

In the presence of a single WDV strain in a cereal growing area, only the preferred host of this viral strain is submitted to an infection risk. This obviously leads to the description of WDV-B in barley fields and of WDV-W in wheat fields. However, larvae as well as winged adult leafhoppers have high dispersal abilities [53]. Consequently, from egg hatching to adult death, leafhoppers are likely to visit numerous healthy and WDV-infected plants from wild and cultivated compartments. Thus, leafhoppers can sequentially acquire isolates of the two WDV strains. Indeed, a previous autumnal (fall) sampling of 903 *P. alienus* individuals (survey carried out in September 2012 on nine French departments) revealed that among the viruliferous *P. alienus* (i.e., 56.2% of caught leafhoppers), the prevalence of insects viruliferous for both WDV-W and WDV-B strains can reach up to 35% (Figure S3). According to the present work, such co-infected leafhoppers should enable the infection of the non-preferred cultivated hosts of each WDV strain. However, the prevalence of co-infected leafhoppers and the rate of co-infected plants by such leafhoppers are relatively low (at least in our field and experimental conditions, respectively). Thus, the presence of WDV-W isolates in barley and WDV-B isolates in wheat is likely to be infrequent in fields.

With its original experimental design based on sequential acquisition/transmission steps and alternate host transfers, this work allows us to conclude on the barley/wheat host specificity of the WDV strains. Indeed, data produced in this work clearly show that wheat and barley are not hosts for WDV-B (isolate b1) and WDV-W (isolate w1), respectively, but each isolate can infect its non-host plant in the presence of the other viral isolate. It is now obvious that the similar ability of *P. alienus* to transmit the two WDV strains, the host specificity of WDV-b1 and WDV-w1 isolates, and the transcomplementation phenomenon observed in co-inoculated plants, can explain most of the contradictory literature reports on the WDV host range. Future work may address the fate of the observed co-infections from the plant to the landscape scales through the vector, including both the within-plant dynamics of the co-infecting viruses and the transmission efficiency of each WDV strain from co-infected plants, as well as the outcome of sequential—rather than simultaneous—inoculations of the two WDV strains.

Indeed, these elements are necessary to predict the epidemiological and economic consequences of the interactions between two viral strains in a landscape [54] comprising wheat and barley fields.

## Supplementary Materials

The following are available online at https://www.mdpi.com/1999-4915/12/1/34/s1.
