# Supplementary material for: Functional Transcomplementation between Wheat Dwarf Virus Strains in Wheat and Barley
Source: Viruses. 2019 Dec 28;12(1):34. doi: 10.3390/v12010034 (PMC7019965; doi:10.3390/v12010034)
Supplement: Supplementary file 1 [file viruses-12-00034-s001.pdf]

viruses

MDPI

Supplementary Materials:

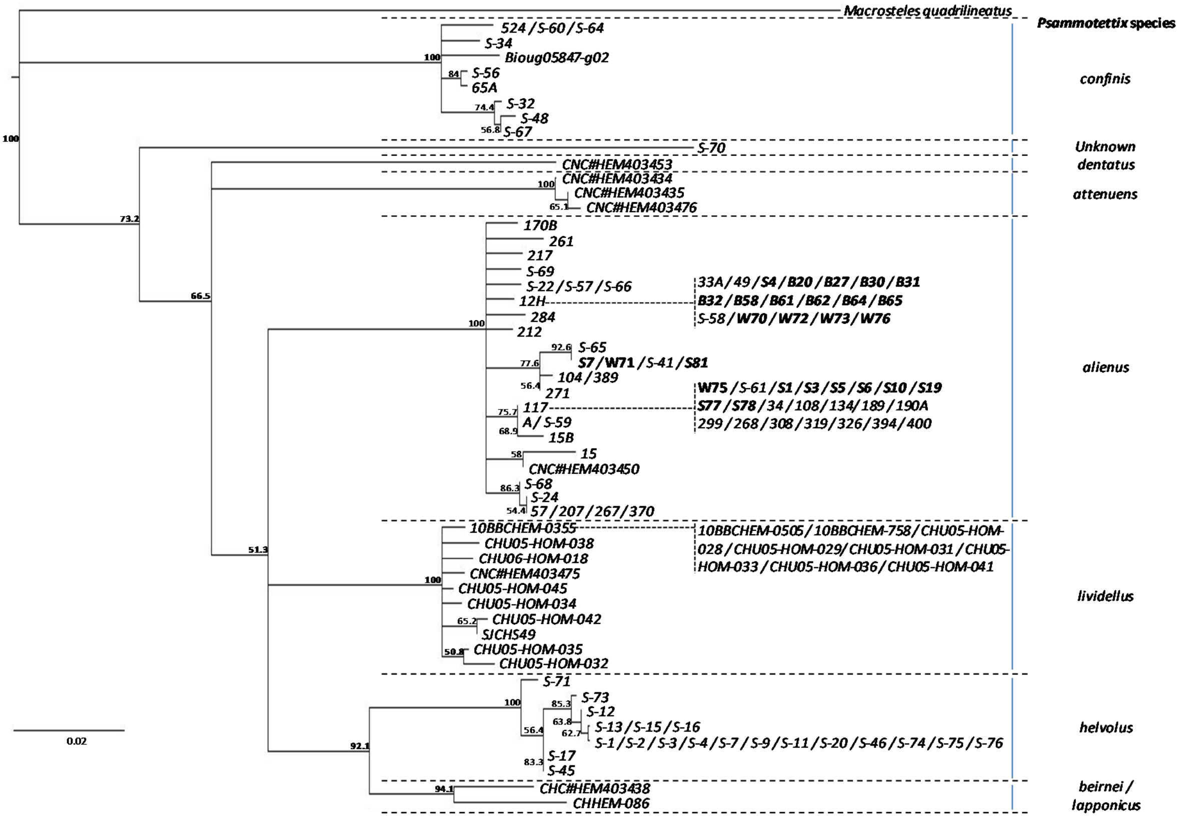

Figure S1. Phylogenetic tree obtained from alignments of 442 nucleotides of cytochrome oxidase I (COI) sequences. *Macrosteles quadrilineatus* COI sequence (GenBank accession number: EU981892.1) was used as outgroup to root the tree. The phylogenetic tree was constructed using the neighbour-joining method implemented into Geneious (Biomatters) with the Tamura and Nei nucleotide substitution model (Tamura & Nei, 1993). Bootstrap percentages obtained with 1000 samples are reported on the branches. The scale bar represents the genetic distance (number of substitutions per nucleotide). Individuals from the large plastic cage rearing systems (PRS) are presented in bold. Leafhoppers sampled from virus-free, viruliferous WDV-w1 and viruliferous WDV-b1 PRS start with 'S', 'W' and 'B', respectively. COI sequences from *Psammotettix* retrieved from GenBank are italicized. Accession numbers are listed in Table S3.

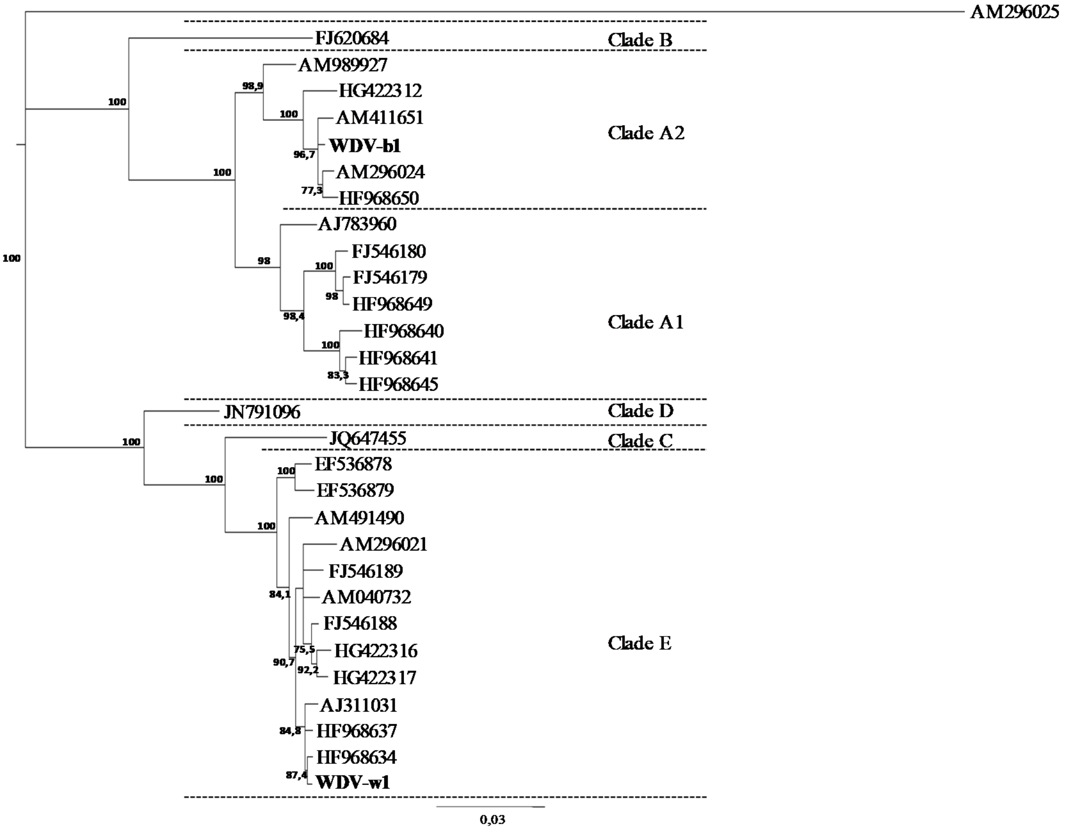

Figure S2. Phylogenetic tree obtained from alignments of WDV full-length sequences. Oat dwarf virus (GenBank accession n° AM296025) was used as outgroup to root the tree. Phylogenetic tree was constructed using neighbour-joining method implemented into Geneious (Biomatters) with the Tamura and Nei nucleotide substitution model (Tamura & Nei, 1993). Bootstrap percentages obtained with 1000 samples are reported on the branches. The scale bar represents the genetic distance (number of substitutions per nucleotide). WDV sequences used to build the tree, selected according to Schubert *et al.* (2014), were retrieved from databases. The two isolates used in the work (WDV-w1 and WDV-b1) are in bold. Clades A1, A2 and B group isolates from WDV barley strain and clades C, D and E group isolated from WDV wheat strain (according to Schubert et al., 2014).

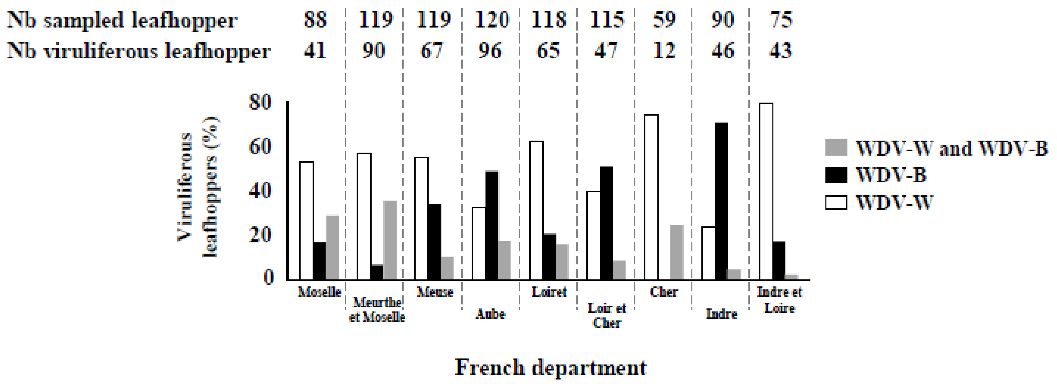

Figure S3. Characterization of WDV strains present in viruliferous *P.alienus* leafhoppers sampled in cereal fields located in 9 French department (up to 4 fields per department) were characterized using the barley- and the wheat- strain-specific PCRassays.

Table S1. Reports of wheat dwarf disease

Area	Country	Reference*
Europe	Hungary	Bisztray et al., 1989
	France	Bendahmane et al., 1995
	Sweden	Lindsten and Lindsten, 1999
	Germany	Huth, 2000
	Poland	Jezewska J. 2001
	Finland	Lemmetty and Huusela-Veistola, 2005
	Spain	Achon and Serrano, 2006
	Bulgaria	Tobias et al., 2009
	Ukraine	Tobias et al., 2011
	United Kingdom	Schubert et al., 2014
	Austria	Schubert et al., 2014
	Slovenia	Marn & Mavric plesko 2017
Middle-East	Turkey	Koklu et al., 2007
	Iran	Behjatnia et al., 2011
Africa	Tunisia	Najar et al., 2000
	Zambia	Kapooria and Ndunguru, 2004
Western-Asia	Syria	Ekzayez et al., 2011
Asia	China	Xie et al., 2007

*: references: Achon, & Serrano, 2006. *Plant Disease, 90*(7), 970-970; Behjatnia et al., 2011. *Australasian Plant Pathology, 40*(1), 12-19; Bendahmane et al., 1995. *Phytopathology, 85*(11), 1449-1455; Bisztray et al., 1989. *Journal of Plant Diseases and Protection, 96*(5), 449-454; Ekzayez et al., 2011. *Plant Disease, 95*(1), 76-76; Huth, 2000. *Journal of Plant Diseases and Protection, 107*(4), 406-414; Jezewska, 2001. Phytopath Polonica 21: 93–100; Kapooria & Ndunguru, 2004. *Bulletin OEPP, 34*(3), 413-419 ; Koklu et al., 2007. *Virus Genes, 34*(3), 359-366; Lemmetty & Huusela-Veistola, 2005. *Plant Disease, 89*(8), 912-912; Lindsten & Lindsten, 1999. *Journal of Plant Diseases and Protection, 106*(3), 325-332; Marn & Mavric plesko, 2017. Plant Disease. 101. 10.1094/PDIS-11-16-1642-PDN; Najar et al., 2000. *Phytopathologia Mediterranea* 39, 423–32; Schubert et al., 2014. *Virus Genes, 48*(1), 133-139; Tobias et al., 2009. *Cereal Research* Cereal Research Communications, 37(2), 237-242; Tobias et al., 2011. *Polish Journal of Microbiology, 60*(2), 125-131; Xie et al., 2007. *Plant Disease* 91, 111.

Table S2. Primers used to amplify the WDV genome

Primer name	Sequence (5' – 3')	Nucleotide position*	Amplified region*
WDV_23F	GCGCACTCGGCTTTTCGTG	23-41	23-843
WDV_824R	CAGCAGATTCCAAGGCATCG	843-824	
WDV_417F	GGTGACCAACAAGGACTCCC	417-436	417-1374
WDV_1356R	AGTGACTGTCCTAGCGCGG	1374-1356	
WDV_824F	CGATGCCTTGGAATCTGCTG	824-843	824-1815
WDV_1795R	TGGATGGACGATTATTCCAGG	1815-1795	
WDV_1356F	CCGCGCTAGGACAGTCACT	1356-1374	1356-2221
WDV_2201R	CAAAGACTGCAACCAAGTTCG	2221-2201	
WDV_1795F	CCTGGAATAATCGTCCATCCA	1795-1815	1795-2750/1--41
WDV_23R	CACGAAAAGCCGAGTGCGC	41-23	
WDV_2201F	CGAACTTGGTTGCAGTCTTTG	2201-2221	2201-2750/1-436
WDV_417R	GGGAGTCCTTGTTGGTCACC	436-417	

*: Nucleotide positions according to WDV-Enk1 isolate (Accession n° AJ311031)

Table S3. Accession numbers of the COI sequences

This study		Retrieved from GenBank			
Leafhopper	Accession n°	Leafhopper	Accession n°	Leafhopper	Accession n°
B20	KT378541	A	KT378496	65A	KT378512
B27	KT378542	S-01	KT378551	34	KT378507
B30	KT378543	S-02	KT378552	117	KT378498
B31	KT378544	S-03	KT378553	108	KT378514
B32	KT378545	S-04	KT378554	134	KT378500
B58	KT378546	S-07	KT378555	49	KT378509
B61	KT378547	S-09	KT378556	57	KT378511
B62	KT378548	S-11	KT378557	15	KT378502
B64	KT378549	S-12	KT378582	104	KT378497
B65	KT378550	S-13	KT378583	170B	KT378503
W70	KT378576	S-15	KT378584	190A	KT378504
W71	KT378577	S-16	KT378585	267	KT378519
W72	KT378578	S-17	KT378586	268	KT378520
W73	KT378579	S-20	KT378558	271	KT378521
W75	KT378580	S-22	KT378587	284	KT378522
W76	KT378581	S-24	KT378588	299	KT378523
S1	KT378532	S-32	KT378589	308	KT378524
S3	KT378533	S-34	KT378590	319	KT378525
S4	KT378534	S-41	KT378559	326	KT378526
S5	KT378535	S-45	KT378560	370	KT378527
S6	KT378536	S-46	KT378561	389	KT378528
S7	KT378539	S-48	KT378562	400	KT378529
S10	KT378530	S-56	KT378591	394	KT378508
S19	KT378531	S-57	KT378592	*CHU06-HOM-018*	KR032827.1
S77	KT378537	S-58	KT378563	*CHU05-HOM-028*	KR044984.1
S78	KT378538	S-59	KT378593	*CHU05-HOM-029*	KR045265.1
S81	KT378540	S-60	KT378564	*CHU05-HOM-031*	KR034629.1
		S-61	KT378565	*CHU05-HOM-032*	KR042346.1
		S-64	KT378566	*CHU05-HOM-033*	KR042177.1
		S-65	KT378594	*CHU05-HOM-034*	KR040121.1
		S-66	KT378595	*CHU05-HOM-035*	KR033388.1
		S-67	KT378567	*CHU05-HOM-036*	KR041908.1
		S-68	KT378568	*CHU05-HOM-041*	KR044208.1
		S-69	KT378569	*CHU05-HOM-038*	KR035932.1
		S-70	KT378570	*CHU05-HOM-042*	KR039979.1
		S-71	KT378571	*CHU05-HOM-045*	KR036332.1
		S-73	KT378572	*CNC#HEM403434*	KR036873.1
		S-74	KT378573	*CNC#HEM403435*	KR041933.1
		S-75	KT378574	*CNC#HEM403438*	KR043152.1
		S-76	KT378575	*CNC#HEM403450*	KR038969.1
		189	KT378515	*CNC#HEM403453*	KR040427.1
		207	KT378516	*CNC#HEM403475*	KR035570.1
		212	KT378517	*CNC#HEM403476*	KR036090.1
		217	KT378505	*10BBCHEM-0355*	KR041223.1
		261	KT378518	*10BBCHEM-0505*	KR043953.1
		33A	KT378506	*10BBCHEM-0758*	KR031005.1
		15B	KT378501	*CHHEM-086*	KR040868.1
		12H	KT378499	*SJCHS49*	KR036014.1
		524	KT378510	*Bioug05847-g02*	KR030552.1

Table S4. Number of plants used in transmission experiments

Virus	IAP (h)	Insect type	Number of inoculated plants per replicate									
WDV-w1*	24	M	9	9	9	10	10	10	10	10	9	10
		F	9	10	9	10	10	10	10	10	10	10
		L	10	10	9	10	10	10	10	10	10	10
	120	M	7	9	7	9	10	10	8	10	9	8
		F	8	9	9	10	7	10	8	9	9	8
		L	9	10	8	10	10	9	10	10	9	9
WDV-b1*	24	M	20	20	20	18	18					
		F	20	19	20	17	19					
		L	18	19	20	20	18					
	120	M	12	17	19	14	15					
		F	18	19	19	15	19					
		L	18	17	20	20	18					

IAP: Inoculation access period; M: Male; F: Female; L: Larvae; WDV-w1 and WDV-b1: wheat and barley strains of *Wheat dwarf virus,* respectively.
* Each insect type/replicate combination was performed using 10 and 20 plants for WDV-w1 and WDV-b1, respectively. Plants with dead insects at the end of the IAPs or with larvae that became adult during the IAP were removed from the data set.

Table S5. Number of plants used in transmission experiments

HAP	Test plant	Rank of test plant in HAP	Inoculated plants per replicate*					
A	Wheat	3rd	10	9	8	8	8	9
	Barley	4th	10	9	8	6	7	7
B	Wheat	4th	7	10	8	8	7	8
	Barley	3rd	7	10	8	8	7	8
C	Wheat	4th	10	9	9	8	6	10
	Barley	3rd	10	9	9	8	6	10
D	Wheat	3rd	7	6	9	8	10	10
	Barley	4th	7	6	9	7	10	10
E	Wheat	3rd	9	10	6	6	7	5
	Barley	4th	9	10	6	6	6	5
F	Wheat	4th	10	8	7	7	9	3
	Barley	3rd	10	8	7	7	9	3
G	Wheat	3rd	7	/	3	6	6	9
	Barley	4th	7	/	3	6	6	7
H	Wheat	4th	7	10	2	5	9	4
	Barley	3rd	7	10	3	5	9	5

* Each replicate combination was performed using 10 plants. Plants with dead insects at the end of the IAP were removed from the data set.

Table S6. Homology between WDV sequences

Sequence analysed	Full length genome	Open reading frames and intergenic regions					
		MP	CP	Rep	RepA	LIR	SIR
WDV-w1 vs. WDV-enk1	99,3	99,6	99,5	99,2	99,1	99	98,8
WDV-b1 vs. WDV-BaW1	98,4	100	99,1	99,6	99,4	92,9	99,4
WDV-w1 vs. WDV-b1	83,6	85,7	83,1	85,5	87,7	69,3	82,5

MP: movement protein; CP: coat protein; Rep/RepA: replicase; LIR: long intergenic region; SIR: short intergenic region. WDV-enk1 (WDV-W strain member): GenBank accession number AJ311031; WDV-BaW1 (WDV-B strain member): GenBank accession number AM411651; WDV-b1: GenBank accession number MN594281 (this work); WDV-w1: GenBank accession number MN594280 (this work).
